# Assessing hypertension care quality in Brazil: gender, race, and socioeconomic intersection in public and private services, 2013 and 2019 national health surveys

**DOI:** 10.1186/s12913-024-11358-5

**Published:** 2024-08-16

**Authors:** Paulo Victor Cesar de Albuquerque, Elaine Tomasi

**Affiliations:** 1 Post-Graduate Program in Epidemiology, Federal University of Pelotas, City of Pelotas, State of Rio Grande do Sul Brazil; 2 Department of Social Medicine, Faculty of Medicine, Federal University of Pelotas, City of Pelotas, State of Rio Grande do Sul Brazil

**Keywords:** Hypertension, Intersectionality, Health care quality, Health services, Epidemiological surveys

## Abstract

We conducted a cross-sectional study of hypertension care in public and private services, analyzing gender, color, and socioeconomic status. Using data from the 2013 (*n* = 60,202) and 2019 (*n* = 90,846) national health surveys, hypertension prevalence increased from 21.4 to 23.9%. Quality of care declined from 41.7 to 35.4%, particularly in public services, disproportionately affecting low-income Black women. Poisson regression estimated prevalence ratios (PRs), with the lowest adjusted PR for high-quality care among low-income Black women. These findings highlight persistent health inequalities and the urgent need for intersectoral policies to promote health equity.

## Introduction

Hypertension is a chronic condition that, when not diagnosed and treated correctly, can cause damage to target organs such as cardiovascular and kidney diseases, increasing the risk of premature death.[1, 2] A study on global trends in blood pressure showed a 90% increase in the number of adults with hypertension between 1975 and 2015, with a more pronounced rise in low and middle-income countries [3].

Quality assessment in healthcare has become crucial for improving work processes and developing service standards that ensure effectiveness and safety, particularly considering the differences between public and private healthcare services.[4, 5] The National Health Survey (NHS), conducted by the Ministry of Health in partnership with the Brazilian Institute of Geography and Statistics (IBGE), provides crucial data to guide health strategies and assess the quality of hypertension care. The second edition of the survey in 2019 allowed for an analysis of progress over time and identification of ongoing challenges [6].

Studies based on the NHS have revealed sociodemographic inequalities in health, particularly affecting women and Black individuals. However, there has been insufficient exploration of how these social determinants overlap and impact individuals facing multiple adverse historical health factors [2, 7]. Analyzing the intersectionality of these sociodemographic characteristics is crucial to uncovering complex and simultaneous forms of oppression, such as racism and sexism, which are often not identified when examined separately [8]. Despite advances made by Brazil’s unified health system, Sistema Único de Saúde (SUS), in universalizing access to healthcare, significant challenges remain in overcoming barriers to hypertension care, especially those imposed by social determinants [9].

Therefore, this study aimed to evaluate and compare hypertension care in public and private healthcare services, analyzing quality indicators through the intersection of gender, race, and socioeconomic status.

## Methods

### Data Source

We used data from the 2013 and 2019 NHSs. Coordinated by the IBGE in partnership with the Ministry of Health, the NHS is a nationwide population-based cross-sectional survey of the domiciled adult population. The data from both surveys are publicly available on the IBGE’s website (https://www.ibge.gov.br/estatisticas/sociais/saude/9160-pesquisa-nacional-de-saude.html). The original questionnaires used in the surveys are available at https://www.pns.icict.fiocruz.br/wp-content/uploads/2021/02/Questionario-PNS-2013.pdf and https://www.pns.icict.fiocruz.br/wp-content/uploads/2023/06/Questionario_PNS_2019_26062023.pdf.

### Sampling

The participants were selected using three-stage cluster sampling as follows: stratification of primary sample units (PSUs) consisting of one or more randomly selected census tracts with probability proportional to the number of private households; simple random selection of households in each PSU using the latest version of the national address register; and simple random selection of residents aged 18 and over without replacement based on a list of residents drawn up during the interview. [10, 11] For the purposes of this study, we selected respondents aged 18 years and over who answered the questions from module Q of the questionnaire about chronic diseases. The sample included respondents who had been clinically diagnosed with hypertension and had had a check-up in the last 24 months.

### Variables

#### Outcome variable

High-quality health care for people with hypertension was assessed using the following 12 dichotomous questions about treatment and advice received during check-up in the last 24 months (No = 0 / Yes = 1): (i) Were you advised to maintain a healthy diet? (ii) Were you advised to maintain a healthy weight? (iii) Were you advised to cut down on salt? (iv) Were you advised to engage in regular physical activity? (v) Were you advised not to smoke? (vi) Were you advised not to drink in excess? (vii) Were you advised to do regular check-ups with a health professional? (viii) During your last check-up, did the doctor look at the results of the tests from past appointments? (ix) Were you asked to do a blood test? (x) Were you asked to do a urinalysis? xi. Were you asked to do an electrocardiogram? xii. Were you asked to do a cardiac stress test? An overall score of over 10 was deemed to indicate high-quality health care.

#### Exposure variables

The exposure variables were divided into three levels: (1) Context – region (North; Northeast; Midwest; Southeast; South); (2) Sociodemographic characteristics: sex (male; female), skin color (white; black/brown), age (18–29; 30–44; 45–59; and 60 years and over), education level (no education/did not complete primary education; completed primary education; completed secondary education; completed higher education), socioeconomic status using economic classification based on the asset index quintiles as a proxy (1st to 5th, lowest to highest), and dichotomized into low (1st, 2nd and 3rd quintiles) and high (4th and 5th quintiles) socioeconomic status; (3) type of health service (public; private). The variable intersection of sociodemographic factors consisted of sex, skin color, and socioeconomic status.

### Statistical analysis

The descriptive analysis was performed using absolute (n) and relative (%) frequencies for the exposure variables and outcome variable. Prevalence rates were stratified according to type of service (public and private). Bivariate analysis was performed to determine the association between high-quality care (95% CI) and region, sociodemographic characteristics, type of service, and intersection of sex, skin color and socioeconomic status, based on prevalence ratios (PRs) estimated using Poisson regression. Multivariate analysis was then performed including variables that obtained a p-value of < 0.20 in the crude analysis to estimate adjusted PRs with their respective 95% CI. Forward stepwise regression was used to select the variables. The variables education level and economic classification, the latter of which was used in this study as a proxy for socioeconomic status, showed similar trends in both editions of the NHS. The former was therefore removed from multivariate analysis because the variable economic status was one of the items of the intersectionality variable. A p-value of < 0.05 in the final model was adopted as a threshold for association.

Due to the NHS’s complex sampling design, the analysis of the survey data requires prior definition of sampling weights for households and selected residents. Statistical analysis was therefore performed using Stata 15 with the *svy* command (StataCorp. 2017. Stata Statistical Software: College Station, TX: StataCorp LP), which considers the weighting of complex sampling designs.

### Ethical considerations

The data from the two editions of the NHS are publicly available and both surveys were approved by the National Research Ethics Committee, attached to the National Health Council (2013 edition: reference Nº 328.159; 2019 edition: reference Nº 3.529.376).

## Results

A total of 60,202 individuals aged 18 years and over participated in the 2013 survey, compared to 90,846 in the 2019 survey. In 2013, 21.4% (95% CI 20.8–22.0) of the respondents reported having been clinically diagnosed with hypertension, compared to 23.9% in 2019 (95% CI 23.5–24.4).

The sampling distribution was similar in both editions of the survey, with the Southeast accounting for the largest proportion of respondents (47.6% in 2013 and 46.9% in 2019), and the North representing the smallest share (5% in 2013 and 5.2% in 2019). The proportion of women (59.7% in 2013 and 59.4% in 2019) and black/brown people (50.3% in 2013 and 54.8% in 2019) was higher than that of men (40.3% in 2013 and 40.6% in 2019) and white people (49.7% in 2013 and 45.2% in 2019) in both surveys. The following groups also accounted for the highest proportions in their category: people aged 60 years and over (42.7% in 2013 and 48.6% in 2019), people who had completed primary education (45.2% in 2013 and 48.6% in 2019), people who used public services (65.8% in 2013 and 68.4% in 2019), and people who had had a check-up in the last 24 months (77.6% in 2013 and 81.5% in 2019). With regard to economic classification, in 2013, the highest quintile accounted for the highest proportion of respondents (22%) and the lowest quintile represented the lowest share (17.3%), while in 2019 the 2nd income quintile accounted for the highest proportion (24.8%) and the 5th quintile represented the lowest share (15%) (Table 1).

**Table 1:** Distribution of individuals clinically diagnosed with hypertension by region, sociodemographic characteristics, type of service, and length of time since last check-up NHS 2013 (*n* = 12,318) and 2019 (*n* = 23,819)

	2013		2019	
Variable	*n*	%	*n*	%
**Region**				
*North*	1,871	5.0	3,625	5.2
*Northeast*	3,674	24.0	8,261	25.2
*Midwest*	1,635	7.3	2,690	6.6
*Southeast*	3,355	47.6	5,989	46.9
*South*	1,783	15.9	3,254	16.0
**Sex**				
*Female*	7,856	59.7	14,441	59.4
*Male*	4,462	40.3	9,378	40.6
**Skin color**				
*White*	5,301	49.7	9,073	45.2
*Black/brown*	7,016	50.3	14,384	54.8
**Age**				
*18–29*	439	3.4	846	3.3
*30–44*	2,228	17.5	3,219	14.3
*45–59*	4,203	36.4	7,326	33.9
*60 and over*	5,548	42.7	12,428	48.6
**Education level**				
*No education/did not complete primary education*	2,970	21.0	3,281	10.9
*Completed primary education*	5,223	45.2	10,696	48.6
*Completed secondary education*	2,579	21.1	5,967	28.1
*Completed higher education*	1,546	12.7	2,771	12.4
**Economic status**				
*1st quintile (lowest)*	2,860	17.3	6,329	20.2
*2nd quintile*	2,796	20.4	5,775	24.8
*3rd quintile*	2,499	20.1	4,630	21.7
*4th quintile*	2,136	20.2	3,709	18.3
*5th quintile (highest)*	2,027	22.0	3,376	15.0
**Type of health service**				
*Private*	3,482	34.2	5,407	31.6
*Public*	7,392	65.8	12,997	68.4
**Time since last check-up**				
*Less than 2 years*	9,638	77.6	18,495	81.5
*2 years and over*	2,680	22.4	4,055	18.5

The prevalence of high-quality care for people with hypertension decreased from 41.7% in 2013 to 35.4% in 2019 (Table 2).

**Table 2:** Prevalence of high-quality care for people with hypertension who had had a check-up in the last 24 months, NHS 2013 (*n* = 9,638) and 2019 (*n* = 18,495)

	2013		2019	
Variable	*n*	%	*n*	%
*Were you advised to maintain a healthy diet?*	9,638	88.6	18,495	87.2
*Were you advised to maintain a healthy weight?*	9,638	85.1	18,495	84.4
*Were you advised to cut down on salt?*	9,638	91.4	18,495	87.8
*Were you advised to engage in regular physical activity?*	9,638	82.1	18,495	81.7
*Were you advised not to smoke?*	9,638	76.4	18,495	67.3
*Were you advised not to drink in excess?*	9,638	75.7	18,495	66.6
*Were you advised to do regular check-ups with a health professional?*	9,638	88.3	18,495	85.3
*At your last check-up*,* did the doctor look at the results of the tests from past appointments?*	8,185	71.0	16,279	69.5
*Were you asked to do a blood test?*	9,638	82.4	18,495	80.2
*Were you asked to do a urinalysis?*	9,638	71.5	18,495	70.2
*Were you asked to do an electrocardiogram?*	9,638	65.8	18,495	64.8
*Were you asked to do a cardiac stress test?*	9,638	36.3	18,495	33.9
**Quality above the threshold (> 10 points)**	**8**,**185**	**41.7**	**16**,**279**	**35.4**

This reduction was observed across all groups, but was more pronounced in percentage point terms in the Southeast (-7.8) and among men (-7.8), black/brown people (-6.3), people aged 60 years and over (-7.7), people who had only completed primary education (-8.4), people with high socioeconomic status (-5.6), people who used public services (-7.0), and in white men with a high economic status (-8.2) (Table 3).

**Table 3:** Prevalence of high-quality care for people with hypertension who had had a check-up in the last 24 months and absolute prevalence difference by region, sociodemographic characteristics, type of service, and intersection of sex, skin color, and socioeconomic status, NHS 2013 (*n* = 8,185) and 2019 (*n* = 16,279)

	2013		2019		Absolute
**Variable**	**n**	**%**	**n**	**%**	differences
**Region**		p-value: 0.000		p-value: 0.000	
*North*	1,285	36.4	2,511	32.8	-3.6
*Northeast*	2,315	33.7	5,403	27.8	-5.9
*Midwest*	1,041	42.9	1,818	39.7	-3.2
*Southeast*	2,333	46.0	4,260	38.2	-7.8
*South*	1,211	41.4	2,287	37.5	-3.9
**Sex**		p-value: 0.005		p-value: 0.005	
*Female*	5,426	39.5	9,756	33.8	-5.7
*Male*	2,759	45.3	6,523	37.5	-7.8
**Skin color**		p-value: 0.000		p-value: 0.000	
*White*	3,581	45.0	6,273	39.4	-5.6
*Black/brown*	4,603	38.3	9,759	32.0	-6.3
**Age**		p-value:0.001		p-value: 0.012	
*18–29*	276	27.4	353	22.0	-5.4
*30–44*	840	35.0	1,827	32.9	-2.1
*45–59*	2,519	42.9	5,150	37.0	-5.9
*60 and over*	4,550	43.1	8,949	35.4	-7.7
**Education level**		p-value: 0.000		p-value: 0.000	
*No education/did not complete primary education*	1,996	31.3	2,305	23.3	-8.0
*Completed primary education*	3,477	40.1	7,395	31.7	-8.4
*Completed secondary education*	1,693	48.6	3,909	41.2	-7.4
*Completed higher education*	1,019	53.7	1,917	50.3	-3.4
**Economic status**		p-value: 0.000		p-value: 0.000	
*1st quintile*	1,823	26.2	4,229	21.7	-4.5
*2nd quintile*	1,840	35.6	4,002	31.8	-3.8
*3rd quintile*	1,661	41.6	3,175	36.1	-5.5
*4th quintile*	1,496	45.1	2,529	42.2	-2.9
*5th quintile*	1,365	55.1	2,344	49.5	-5.6
**Type of health service**		p-value: 0.000		p-value: 0.000	
*Private*	2,679	53.5	4,820	50.2	-3.3
*Public*	5,438	35.5	10,864	28.5	-7.0
**Intersectionality**		p-value: 0.000		p-value: 0.000	
*Male*,* white and high SES*	601	57.5	1,128	49.3	-8.2
*Male*,* white and low SES*	647	36.8	1,487	32.9	-3.9
*Male*,* black and high SES*	511	51.3	903	46.0	-5.3
*Male*,* black and low SES*	1,000	34.4	2,914	30.5	-3.9
*Female*,* white and high SES*	967	49.0	1,478	45.4	-3.6
*Female*,* white and low SES*	1,366	36.4	2,180	33.4	-3.0
*Female*,* black and high SES*	782	42.9	1,280	41.0	-1.9
*Female*,* black and low SES*	2,310	33.6	4,662	27.4	-6.2

**Fig. 1:**
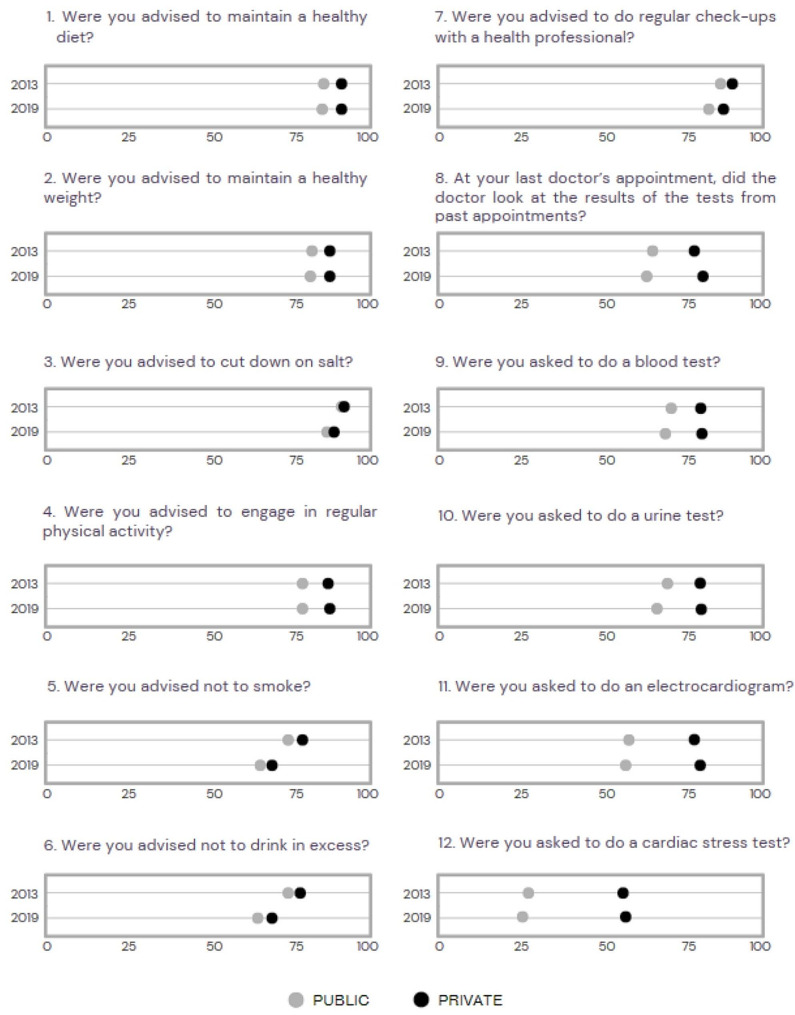
Overall proportions for each item of the score for the quality of hypertension care for individuals who had had a check-up in the last 24 months by a service type, NHS 2013 (*n* = 8,185) and 2019 (*n* = 16,279)

Among public and private healthcare services, a significant quality predominance was observed in private services regarding each component comprising the quality score. Additionally, from 2013 to 2019, there was a substantial decline in the quality of public services concerning most of these components, while private services experienced a significant increase in quality in most items, resulting in the widening disparity between these two types of healthcare services (Fig. 1).

The results of the crude analysis show that, in 2013, the PR of high-quality care was higher in the Southeast [1.26 (1.08–1.47)] and in people aged 60 years and over [1.57 (1.14–2.18)], and lower in people who used public services [0.66 (0.61–0.73)] and black women with low socioeconomic status [0.59 (0.50–0.69)]. In 2019, PR was higher in the Midwest [1.21 (1.06–1.37)] and among people aged between 45 and 59 years [1.68 (1.18–2.41)], and lower in people who used public services [0.57 (0.52–0.61)] and black women with low socioeconomic status [0.56 (0.48–0.64)] (Table 4).

**Table 4:** Crude and adjusted prevalence ratios (PR) for high-quality health care for people with hypertension who had had a check-up in the last 24 months by region, age, type of health service, and patterns of intersectionality. NHS 2013 (*n* = 8,185) and 2019 (*n* = 16,279)

	2013	2013	2019	2019
Variable	PR (95% CI)	PR (95% CI)	PR (95% CI)	PR (95% CI)
	Crude	Adjusted	Crude	Adjusted
**Region**	p-value: 0.000		p-value: 0.000	
*North*	1.00		1.00	
*Northeast*	0.93 (0.78–1.10)		0.85 (0.76–0.95)	
*Midwest*	1.18 (1.00–1.39)		1.21 (1.06–1.37)	
*Southeast*	1.26 (1.08–1.47)		1.16 (1.03–1.31)	
*South*	1.14 (0.96–1.35)		1.14 (1.01–1.29)	
**Age**	p-value: 0.000	p-value: 0.003	p-value: 0.091	
*18–29*	1.00	1.00	1.00	
*30–44*	1.28 (0.91–1.79)	1.29 (0.91–1.81)	1.50 (1.04–2.16)	
*45–59*	1.56 (1.13–2.16)	1.55 (1.11–2.16)	1.68 (1.18–2.41)	
*60 and over*	1.57 (1.14–2.18)	1.60 (1.15–2.22)	1.61 (1.13–2.30)	
**Type of health service**	p-value: 0.000	p-value: 0.000	p-value: 0.000	p-value: 0.000
*Private*	1.00	1.00	1.00	1.00
*Public*	0.66 (0.61–0.73)	0.74 (0.67–0.81)	0.57 (0.52–0.61)	0.64 (0.58–0.70)
**Patterns of intersectionality**	p-value: 0.000	p-value: 0.052	p-value: 0.000	p-value: 0.090
*Male*,* white and high SES*	1.00	1.00	1.00	1.00
*Male*,* white and low SES*	0.64 (0.53–0.78)	0.72 (0.58–0.88)	0.67 (0.57–0.78)	0.94 (0.78–1.12)
*Male*,* black and high SES*	0.89 (0.74–1.08)	0.98 (0.81–1.19)	0.93 (0.80–1.09)	1.05 (0.90–1.24)
*Male*,* black and low SES*	0.60 (0.59–0.74)	0.73 (0.59–0.92)	0.62 (0.53–0.72)	0.95 (0.79–1.14)
*Female*,* white and high SES*	0.85 (0.72–1.01)	0.88 (0.75–1.04)	0.92 (0.80–1.06)	0.97 (0.85–1.12)
*Female*,* white and low SES*	0.63 (0.53–0.75)	0.70 (0.59–0.84)	0.68 (0.59–0.78)	0.96 (0.80–1.14)
*Female*,* black and high SES*	0.75 (0.62–0.89)	0.82 (0.68–0.98)	0.83 (0.71–0.97)	0.96 (0.82–1.12)
*Female*,* black and low SES*	0.59 (0.50–0.69)	0.72 (0.60–0.87)	0.56 (0.48–0.64)	0.86 (0.73–1.02)

In the adjusted analysis, in 2013, the PR continued to be higher among people aged 60 years and over [1.60 (1.15–2.22)] and lower in people who used public services [0.74 (0.67–0.81)]. With regard to intersectionality, the results reveal a pattern whereby people with low socioeconomic status showed lower PR, regardless of color or sex. In 2019, the adjusted prevalence ratios (PRs) indicated that people who used public services continued to have the lowest PR for high-quality health care [0.64 (0.58–0.70)]. With regard to intersectionality, black women with low socioeconomic status continued to have the lowest PR [0.86 (0.73–1,02)] for high-quality health care, although without statistical significance (Table 4).

## Discussion

In line with previous research, the findings of the present study reveal that the prevalence of hypertension in Brazil rose between 2013 and 2019 [2, 6, 12, 13]. The data from the two surveys show that there was no significant change in the profile of people with hypertension during the study period. These findings suggest that policies to promote equity in health care among people with hypertension have not been effective. Health actions aimed at controlling hypertension need to be planned, ongoing, integrated and, above all, subjected to quality assessment[14].

Despite the relationship between health and access to and utilization of health services, the latter often fail to meet quality standards for the diagnosis and control of diseases.^5^ The use of protocols for hypertension care can improve service delivery and quality control and facilitates the identification of factors that influence care quality and outcomes. [15] Despite broad consensus about the importance of clinical guidelines and protocols, their use remains limited, especially when it comes to the implementation of actions based on the risk stratification of a given population group, posing a barrier to improving access and achieving better care outcomes based on current scientific knowledge. [16, 17] It is worth noting that the questions used in the NHS to assess quality of care are derived from protocols and official guidelines.

Access to and utilization of health care services are complex and multidimensional concepts. People who have access to a greater range of sources of care are able to access a more diverse set of preventive and therapeutic services. [18] Our findings show that people who used private services reported higher quality of care; however, most of the respondents used public services. Studies show that patients who use exclusively public services tend to be more vulnerable, especially among the black population with low socioeconomic status living in the North and Northeast [19].

Quality of care declined between 2013 and 2019 across all sociodemographic groups, regions, and types of service. Health inequalities linked to gender, skin color, and socioeconomic status persisted, reflecting deep-rooted inequality in Brazilian society. [7, 20] Previous studies have shown that people with a higher level of education tend to have improved access to health services and receive better quality care. Level of education can also influence health literacy and patient understanding of recommendations and advice provided by health professionals [21].

The decline in quality of hypertension care was more pronounced in public services. Given that most appointments in public services take place in primary care services, the point of entry to the health system, these findings may be attributed to recent changes in the National Primary Health Care Policy (PNAB), which altered the composition of health teams and permitted a reduction in staff and working hours. In view of increasing demand for public services due to the impoverishment of the population, the new PNAB has increased the burden on health professionals, in turn affecting the quality of care. Another factor that may have contributed to the decline in the overall performance of public services is the passing of Constitutional Amendment 95 in 2016 imposing a ceiling on health spending, precisely in the middle of the study period [5]

The results of the NHS highlight the urgent need for research to advance understanding of the complex relationship between social inequalities and chronic disease and quality of health care. In this direction, the findings of the present study suggest that greater emphasis is needed on the implementation of intersectoral policies. In this sense, the use of the concept of intersectionality in policy-making helps policymakers to understand the multiple social determinants of health [25].

Women and men should therefore not be assessed as homogenous groups without taking into account factors such as race/skin color. Women face various social restrictions – including lower salaries and unpaid domestic work – limiting the amount of time available for formal employment, education, and leisure, and impacting health care. In addition, the intersection of sexism and racism, both interlocking forms of oppression, hierarchization, and social exclusion, disproportionately affects black women [23].

The analysis of black women’s health within the context of Brazil’s history requires an intersectional approach that explores the intersection of different categories of analysis: gender, race/color, and socioeconomic status. Intersectionality is a key theoretical and methodological framework for understanding social dynamics that impact women’s lives, enabling researchers to capture the structural consequences and dynamics of interaction between two or more forms of subordination [24].

Social reality in Brazil is characterized by major racial disparities in living conditions, health, and mortality, as well a belief in the superiority of economically and politically dominant groups. [25] These beliefs are ingrained in both common sense and academic knowledge, and bringing to light these inequalities – particularly in public health systems – is a subversive and necessary activity [24, 26].

Black women tend to have a lower level of education and income than other groups and are more likely to have more taxing jobs with longer working hours and little social recognition and a heavier domestic work load. [27] At the confluence of these factors lies the three-fold invisibility and violence to which black women are subjected to, as they are the only group attacked on three fronts: misogyny, racism, and poverty. [28–30] Promoting equity in health care quality is therefore essential for improving well-being, preventing the perpetuation of health disparities, and minimizing the disproportional effects of health inequality on disadvantaged groups [6].

First, the cross-sectional nature of the research design limits our ability to determine temporality and causal relationships between variables. Second, the study may be subject to information and recall biases, particularly regarding the quality score items reported by participants, which could affect the accuracy of responses. Third, there may be specification bias in the variables chosen to compose the quality score, as other variables not included could influence the results. Additionally, the samples from the 2013 and 2019 National Health Surveys (NHS) had different sizes, potentially introducing variations in data comparison over time. Furthermore, it was not possible to stratify clinical risk for hypertension, such as identifying cardiovascular factors. Another limitation is the variable “sex” as the relevant field in the IBGE questionnaires only has two response options (male and female), disregarding gender identity, which is particularly important for constructing the intersectionality variable.

The differences between raw and adjusted prevalence ratios (PRs) highlight the influence of confounding variables on the quality of care. For instance, the raw PRs suggested a higher likelihood of high-quality care in certain groups, but once adjusted for factors like socioeconomic status, the disparities became more pronounced. This indicates that the initial observed differences in care quality might be partly attributed to underlying sociodemographic factors. Adjusting for these factors provides a clearer picture of the true disparities in care quality, underscoring the need for targeted interventions.

The results suggest an urgent need for intersectoral policies that address the multiple forms of oppression exacerbating health inequalities. Implementing specific clinical guidelines and protocols, along with continuous education for health professionals, can improve care quality and reduce the identified disparities. Additionally, public policies must be designed considering the intersectionality of social determinants of health, ensuring that interventions are effective for the most vulnerable groups.

In conclusion, this study highlights the importance of intersectional approaches in analyzing health inequalities and formulating policies. Recognizing the complex interactions between gender, race/color, and socioeconomic status is crucial for promoting health care equity and improving outcomes for individuals with hypertension.

## Data Availability

We used data from the 2013 and 2019 NHSs. Coordinated by the IBGE in partnership with the Ministry of Health. The data from both surveys are publicly available on the IBGE’s website (www.ibge.gov.br/estatisticas/sociais/saude/9160-pesquisa-nacional-de-saude.html). The original questionnaires used in the surveys are available at www.icict.fiocruz.br/questionarios/.
